# PLA2G2F/lysoplasmalogen axis links epidermal lipid metabolism to type 2 inflammation and itch in atopic dermatitis

**DOI:** 10.3389/fimmu.2026.1880255

**Published:** 2026-09-07

**Authors:** Yoshimi Miki, Natsumi Higashisaka, Honami Inubushi, Niki Hirabayashi, Kanji Watanabe, Saho Fukui, Masayoshi Onitsuka, Chiaki Fukaura, Mariko Ogawa-Momohara, Kana Tanahashi, Yuki Nagasaki, Takuya Takeichi, Masashi Akiyama, Makoto Murakami, Kei Yamamoto

**Affiliations:** 1 Division of Bioscience and Bioindustry, Graduate School of Technology, Industrial and Social Sciences, Tokushima University, Tokushima, Japan; 2 Graduate School of Biomedical Sciences, Pharmaceutical Sciences, Tokushima University, Tokushima, Japan; 3 Institute of Photonics and Human Health Frontier, Tokushima University, Tokushima, Japan; 4 Department of Dermatology, Nagoya University Graduate School of Medicine, Nagoya, Japan; 5 Laboratory of Microenvironmental Metabolic Health Sciences, Center for Disease Biology and Integrative Medicine, Graduate School of Medicine, The University of Tokyo, Tokyo, Japan; 6 Division of Physiological Chemistry and Metabolism, Department of Biochemistry and Molecular Biology, Graduate School of Medicine, The University of Tokyo, Tokyo, Japan; 7 Nagoya University Institute for Advanced Research, Nagoya, Japan; 8 Department of Dermatology, Shin-yurigaoka General Hospital, Kawasaki, Japan; 9 AMED-CREST, Japan Agency for Medical Research and Development, Tokyo, Japan; 10 PRIME, Japan Agency for Medical Research and Development, Tokyo, Japan

**Keywords:** atopic dermatitis, itch, lipidomics, lysoplasmalogen, phospholipase A_2_

## Abstract

Atopic dermatitis is a chronic inflammatory skin disease characterized by type 2 immune responses and severe itching; however, the molecular mechanisms linking lipid metabolism to epithelial–immune signaling remain incompletely understood. Herein, we show that group IIF secreted phospholipase A_2_ (PLA2G2F), which is induced by type 2 cytokines and selectively generates plasmalogen-derived lysophosphatidylethanolamine (lysoplasmalogen; P-LPE) in keratinocytes, contributes to the aggravation of atopic dermatitis and itching. Genetic deletion of *Pla2g2f* attenuated IL-33 expression in keratinocytes and reduced epidermal hyperplasia, serum IgE levels, type 2 inflammation and scratching behavior, without directly affecting neuronal structure and function, in a mouse model of atopic dermatitis. Topical application of a secreted PLA_2_ inhibitor or forcible enzymatic degradation of P-LPE suppressed the itch response in wild-type mice, whereas exogenous P-LPE partially restored scratching behavior in *Pla2g2f*-deficient mice. Importantly, P-LPE levels were significantly elevated in the stratum corneum of patients with atopic dermatitis and positively correlated with disease severity. Collectively, the present study highlights that the PLA2G2F/P-LPE axis, originally identified in psoriasis, is a key regulator that connects epidermal lipid metabolism to IL-33–mediated type 2 inflammation and itching, suggesting that this pathway could be a novel therapeutic target and biomarker of this disease.

## Introduction

Atopic dermatitis is a common, chronic relapsing inflammatory skin disease characterized by dry skin and pruritic eczematous lesions, affecting 10–20% of individuals in developed countries. Histologically, atopic dermatitis is associated with epidermal hyperplasia and the infiltration of inflammatory cells, including lymphocytes, eosinophils, and mast cells. The pathogenesis of atopic dermatitis is driven by the interplay among type 2 immune dysregulation, skin barrier dysfunction, and pruritus, which mutually amplify each other. Th2 cytokines, such as interleukin (IL)-4, IL-13, and IL-31, impair epidermal barrier function and directly induce itching, leading to scratching-mediated barrier disruption and further immune activation via epithelial-derived alarmins, including IL-33 and thymic stromal lymphopoietin (TSLP) (1–3), highlighting the importance of epithelial–immune–neuronal interactions driving the itch–scratch cycle in atopic dermatitis (4). Current therapeutic strategies, including topical corticosteroids, calcineurin inhibitors, and JAK inhibitors, as well as systemic biologics targeting type 2 cytokines (e.g., anti–IL-4Rα and –IL-31R antibodies), have substantially improved disease management. However, these treatments are sometimes associated with limitations such as adverse effects, high medical costs, and non-responders. Therefore, there remains an unmet need to identify novel therapeutic targets that more effectively interrupt the pathological crosstalk among epithelial, immune, and neuronal pathways in atopic dermatitis.

Lipids are major structural and functional components of the epidermis that play essential roles in maintaining skin barrier integrity (5–9). Beyond their structural functions, lipids act as bioactive mediators that regulate inflammation and cellular signaling in the skin (10). Accumulating evidence has indicated that lipid mediators critically regulate these pathogenic processes in atopic dermatitis. In particular, prostaglandins (PGs) and leukotrienes (LTs) such as PGE_2_, LTB_4_, and cysteinyl LTs (LTC_4_, D_4_ and E_4_) which are produced from arachidonic acid (ω6 20:4), have been detected in atopic dermatitis lesions and modulate type 2 immune responses (11–16), epithelial barrier function (17–19), and pruritus (20–23) through complex and context-dependent mechanisms. In contrast, ω3 fatty acid–derived specialized pro-resolving mediators, such as resolvins and protectins, actively terminate inflammation and promote tissue homeostasis (24). Patients with atopic dermatitis exhibit reduced levels of ω3-derived specialized pro-resolving mediators together with a predominance of ω6-derived pro-inflammatory lipid mediators, suggesting an impaired resolution of inflammation (25). In addition to these fatty acid-derived lipid mediators, lysophospholipid-derived mediators, such as lysophosphatidic acid (LPA), which is produced from lysophosphatidylcholine (LPC) by autotaxin, have also been implicated in skin pathophysiology (26). These lipid mediators regulate the key aspects of atopic dermatitis pathology, including cytokine production, immune cell recruitment, skin barrier dysfunction, tight junction collapse, and pruriceptive nerve activation.

The generation of these lipid mediators is primarily initiated by the hydrolysis of membrane phospholipids by phospholipase A_2_ (PLA_2_). The PLA_2_ superfamily comprises multiple structurally and functionally distinct enzymes, including secreted PLA_2_s (sPLA_2_s), cytosolic PLA_2_s (cPLA_2_s), Ca^2+^-independent PLA_2_s (iPLA_2_s) and several others, each exhibiting unique substrate specificity, tissue distribution, and biological function (27). Recent studies have implicated several sPLA_2_ isoforms in the exacerbation and resolution of inflammatory diseases (28–31). Group IIF sPLA_2_ (PLA2G2F) is uniquely expressed in epidermal keratinocytes and has emerged as a key regulator of skin lipid metabolism. PLA2G2F promotes the preferential production of plasmalogen-derived lysophosphatidylethanolamine (lysoplasmalogen; P-LPE), thereby contributing to Th17-driven psoriasis, Th1-driven contact dermatitis, and skin cancer through the regulation of epidermal lipid mediated immune signaling (32, 33). These findings suggest that PLA2G2F links epidermal lipid signaling to immune responses in various skin diseases. However, whether the PLA2G2F-dependent lipid pathway is also involved in skin diseases with type 2 inflammation, such as atopic dermatitis, remains unclear. In particular, the role of P-LPE in regulating epithelial–immune crosstalk in Th2-dominant microenvironments is not well defined.

In this study, we investigated the role of PLA2G2F and the underlying lipid metabolism in atopic dermatitis–like inflammation. Using a combination of *in vitro* analyses, an *in vivo* mouse model, lipidomics, and human samples, we demonstrated that the PLA2G2F/P-LPE axis links epidermal lipid metabolism to IL-33–associated type 2 inflammation and itch, thus providing new insights into the integration of lipid metabolism and immune signaling in the pathogenesis of atopic dermatitis and underscoring this lipid pathway as a potential therapeutic target for itch.

## Materials and methods

### Cell culture

Immortalized newborn human epidermal keratinocytes (NHEK/SVTERT3-5, Evercyte) were cultured in HuMedia KG2 Medium (KURABO) at 37 °C under a humidified atmosphere of 7% (v/v) CO_2_ in air. NHEK/SVTERT3–5 cells were transfected with 10 nM human *PLA2G2F* siRNA (Flexi Tube siRNA, Hs_PLA2G2F_2; 5-TACCAGGAACTCTTTGACCAA-3; QIAGEN) or control siRNA (All Stars Negative Control siRNA; QIAGEN) using Lipofectamine RNAiMAX (Invitrogen), in accordance with the manufacturer’s instructions. The cells were then treated with a type 2 cytokine cocktail (IL-4, IL-13, and TSLP (all from Prospec); 10 ng/mL each) for 24 h. The cells were then harvested for gene expression analysis, and their culture supernatants were collected for lipid extraction followed by lipidomic analysis.

### RNA isolation and quantitative RT-PCR

Total RNA was extracted from cells and tissues using TRIzol reagent (Invitrogen). First-strand cDNA synthesis was performed using a High Capacity cDNA Reverse transcription kit (Applied Biosystems). PCR reactions were performed using a Power SYBR Green PCR system (Applied Biosystems) or a TaqMan Gene Expression System (Applied Biosystems) on the Step One Plus Real-Time PCR System (Applied Biosystems). The probe and primer sets used are listed in **Supplementary Tables 1**, **2**, respectively.

### Lipidomics

Mass spectrometry (MS)-based lipidomic analysis was performed according to our published protocol with slight modifications (34). Briefly, for the detection of phospholipids, lipids extracted from the samples by the method of Bligh and Dyer (35) were subjected to electrospray ionization (ESI)-MS analysis using a QTRAP6500 (Sciex) or an LCMS-8040 (Shimadzu) triple quadrupole-linear ion trap hybrid mass spectrometer with a reverse-phase liquid chromatography (LC) (NexeraX2 system, Shimadzu). The samples were injected by an autosampler, applied to a Kinetex C18 column (2.1 x 50 mm, 1.7 μm particle, Phenomenex) coupled to ESI-MS, and separated by a step gradient with mobile phase A (acetonitrile/methanol/water = 1.5:1:1 [v/v/v] containing 5 μM phosphoric acid and 1 mM ammonium formate) and mobile phase B (2-propanol containing 5 μM phosphoric acid and 1 mM ammonium formate) at a flow rate of 0.2 ml/min at 50 °C. Lipids were identified using multiple reaction monitoring (MRM) transition and retention times, and quantification was performed based on the peak area of the MRM transition and the calibration curve obtained with an authentic standard for each compound. As internal standards, 50 pmol *d5*-eicosapentaenoic acid (EPA) (Cayman Chemical) and 18:1-*d7* LPE (Avanti) were added to each sample.

### Animals

*Pla2g2f*^–/–^ mice on a Balb/c background were described previously (32). Balb/c mice were purchased from Japan SLC. The mice were housed in climate-controlled facilities at 23 °C with a 12 h light-dark cycle, with free access to standard laboratory food (CE2, CLEA) and water. All animal experiments were approved by Tokushima University (approval numbers T27-95, T30-118, T2021-99, T2024-82) and conformed to the Japanese Guide for the Use of Laboratory Animals. Mice were anesthetized with isoflurane (Wako) using an inhalation anesthesia vaporizer system (3–5% for induction and 1.5–2% for maintenance in oxygen) when required for experimental procedures. The depth of anesthesia was monitored by assessing respiratory rate and pedal reflexes. For tissue collection and euthanasia, mice were deeply anesthetized with isoflurane followed by cervical dislocation, in accordance with institutional guidelines for animal care and use.

### MC903-induced atopic dermatitis–like skin inflammation model

Atopic dermatitis–like skin inflammation was induced by repeated topical application of MC903 (calcipotriol), as previously described with slight modifications (36). MC903 (Cayman Chemical) was applied at a dose of 0.8 nmol per ear surface, totaling 3.2 nmol for both sides of both ears. Control mice received vehicle (ethanol) alone. Ear thickness and scratching behavior were monitored as indicators of inflammation and pruritus. Ear thickness was measured for each mouse before and after elicitation for various time periods at a predetermined site with a micrometer (Mitsutoyo), and the difference was expressed as ear swelling. During the measurement procedures, mice were lightly anesthetized with isoflurane (2% for maintenance in oxygen). Scratching behavior was evaluated by placing the mice individually in cages and allowing a 30-min acclimation period, followed by 30 min of video recording. The number of scratching bouts directed to the ear with the hind paw was quantified, with one bout defined as a single lifting movement of the hind paw. At the end of the experiments, the mice were euthanized by cervical dislocation, and ear tissues were collected for subsequent analyses. As required for experiments, recombinant *Actinomycete* lysoplasmalogen-specific phospholipase D (LyPls-PLD) (33, 37), FK506 (Wako), LPE(P-18:0) (Avanti), or an sPLA_2_ inhibitor (12e; 2-(3-(2-amino-2-oxoacetyl)-1-benzyl-2-ethyl-1H-6,7-benzoindol-4-yloxy)acetic acid) (38) was topically co-applied with MC903 one day before measurement.

### Histology and immunohistochemistry

Mouse tissues were fixed in 10% (v/v) neutral buffered formalin (Wako) and embedded in paraffin. Sections (5-μm thick) were prepared and stained with hematoxylin and eosin. For immunofluorescence analysis, paraffin-embedded sections were deparaffinized, and the tissue sections were then incubated with 1× BlockAce (DS Pharma Biomedical) in PBS-T for 30 min, followed by three washes with PBS-T (5 min each). Sections were then incubated overnight at 4 °C with rabbit anti–mouse PLA2G2F antibody (39) at a 1:1,000 dilution in 10-fold–diluted BlockAce. After washing three times with PBS-T (5 min each), the sections were incubated with Alexa Fluor 488–conjugated goat anti–rabbit IgG antibody (Molecular Probes; 1:1,000) at 4 °C for 5 h. Nuclei were counterstained using VECTASHIELD Plus Antifade Mounting Medium with DAPI (Vector Laboratories). Fluorescence images were acquired using an all-in-one fluorescence microscope (BZ-X800; Keyence).

### Measurement of serum IgE

For serum collection, mice were deeply anesthetized with isoflurane. Whole blood was collected from the inferior vena cava using a syringe and allowed to clot at room temperature. Sera were obtained by centrifugation at 3,000 x *g* for 10 min at room temperature and stored at −80 °C until use. Serum IgE levels were quantified using a commercially available ELISA kit (Bethyl Laboratories), according to the manufacturer’s protocol.

### Flow cytometry

Cells were prepared from mouse ear skin by enzymatic digestion with 400 U/mL collagenase type II (Worthington) and 0.1 mg/mL DNase I (from bovine pancreas; Sigma) for 60 min at 37 °C with shaking. After the addition of 10 mM EDTA, the cell suspension was passed through a 70-μm cell strainer (Corning). Red blood cells were lysed for 2 min on ice using lysis buffer containing 10 mM Tris-HCl (pH 7.0) and 0.84% (w/v) ammonium chloride. Cells were then washed and stained with a fixable viability dye (Thermo) in PBS for 20 min at room temperature in the dark. After washing with FACS buffer, cells were preincubated with purified rat anti–mouse CD16/CD32 (clone 2.4G2, BD Biosciences) and subsequently stained with fluorochrome-conjugated antibodies against mouse CD45 (clone 30-F11, BioLegend) and TCRβ (clone H57-597, BioLegend). Following surface staining, cells were fixed and permeabilized using Cytofix/Cytoperm solution (BD Biosciences) according to the manufacturer’s instructions and then stained with antibodies against IL-4 (clone 11B11, BioLegend) and IL-13 (clone W17010B, BioLegend). Flow cytometric analysis was performed using FACSVerse (BD Biosciences). Data analysis was performed using the manufacturer’s software (BD FACSuite, BD Biosciences).

### Collection of the stratum corneum from mouse ears using cotton swab

Stratum corneum samples were collected from both sides of mouse ears using cotton swabs pre-moistened with ethanol. During the sampling procedure, mice were lightly anesthetized with isoflurane (2% for maintenance in oxygen). The entire ear surface (both dorsal and ventral sides) was swabbed with moderate pressure five times using a new swab for each collection. The collected samples were placed into microtubes and stored at −80 °C. Lipids were subsequently extracted from the cotton swab samples and subjected to lipidomic analysis.

### DRG isolation

Mice were euthanized by cervical dislocation, and dorsal root ganglia (DRGs) were carefully dissected from the intervertebral foramina. The DRGs were incubated in DMEM/F12 containing 2 mg/mL collagenase type III (Worthington) at 37 °C for 120 min with gentle agitation. Subsequently, the tissues were incubated in 2.5 mg/mL trypsin (Type I, Sigma) in 1.5 ml of Hanks’ balanced salt solution at 37 °C for 15 min. Then, 150 μl of trypsin inhibitor (Sigma; 50 μg/mL) was added, and the cells were gently dissociated by pipetting 20–30 times. The cell suspension was layered onto 30% (v/v) Percoll (GE Healthcare) and subjected to density gradient centrifugation (180 × *g*, 5 min) to separate neurons from non-neuronal cells. The collected neurons were washed twice with DMEM/F12 supplemented with 10% FBS (180 × *g*, 3 min), resuspended in 2 ml of DMEM/F12 containing 10% FBS per tube, and plated onto poly-L-lysine–coated culture dishes for subsequent culture. Neurite outgrowth was observed using an all-in-one microscope (BZ-X800; Keyence).

### Measurement of PLA2G2F enzymatic activity

PLA2G2F-expressing Chinese hamster ovary (CHO) cells were prepared by transiently transfecting with human *PLA2G2F* cDNA in an in-house designed vector, based on a previously described CHO expression system (40). Control cells were generated by transfection with the corresponding empty vector. Enzymatic activity in conditioned medium from PLA2G2F-expressing CHO cells was assessed using a natural membrane assay, as described previously (34). Briefly, total lipids were extracted from MC903-treated ear skin using the method of Bligh and Dyer (35). The resulting lipid fraction was resuspended and sonicated for 5 min in 50 mM Tris–HCl (pH 7.4) containing 2 mM CaCl_2_ to generate membrane mimetics. These preparations were incubated with various concentrations of the culture medium from PLA2G2F-expressing or control CHO cells at 37 °C for 30 min. Following incubation, lipids were mixed with internal standards (10 pmol 18:1-*d7* LPE), extracted, and subjected to lipidomics for the detection of lysophospholipids and free fatty acids. As required for experiments, the conditioned medium was preincubated with the sPLA_2_ inhibitor (12e; 2-(3-(2-amino-2-oxoacetyl)-1-benzyl-2-ethyl-1H-6,7-benzoindol-4-yloxy)acetic acid) (38) at 37 °C for 5 min, after which the enzymatic reaction was initiated by the addition of substrate.

### Human samples

Human stratum corneum samples prepared from volunteers via tape stripping or cotton swab were obtained from Nagoya University (approval number 2018-0310) and Tokushima University (approval numbers 18011 and 24001), following approval by the respective Institutional Research Ethics Committees and with written informed consent obtained from all participants.

For tape-stripping–based collection, a 25 × 60 mm piece of film-masking tape (Teraoka) was applied to the inner forearm, gently pressed, and then removed. This procedure was repeated five times using a new piece of tape for each strip. The collected tape samples were mounted onto a plastic sheet (A4 size; 210 × 297 mm) and stored at −80 °C until analysis.

For cotton swab–based collection, a sterile cotton swab (4.7 × 75 mm; Osaki Medical) was applied to the inner forearm without the use of any solvent or buffer and gently rubbed across the skin surface. The swab was moved back and forth over the same area for 20 strokes to collect superficial stratum corneum components. After collection, each swab was placed into a 15-mL tube and stored at −80 °C until analysis.

Clinical severity was assessed using the Eczema Area and Severity Index (EASI) and the Investigator’s Global Assessment (IGA) according to established scoring systems. EASI scores were calculated based on the extent and severity of eczema (erythema, edema/papulation, excoriation, and lichenification) across four body regions. IGA scores were assigned on a 0–4 scale, with higher scores indicating more severe disease.

### Statistical analysis

The experiments were performed and analyzed in a non-randomized and non-blind manner. Data are presented as mean ± SEM. Statistical analyses were performed using GraphPad Prism 9. Comparisons between two groups were conducted using Student’s *t*-test. For multiple-group comparisons, one-way or two-way ANOVA followed by Tukey’s multiple comparisons test was used, as appropriately. Correlations between lipid levels and clinical parameters were evaluated using Pearson’s and Spearman’s rank correlation coefficient (r) with two-tailed P value.

## Results

### PLA2G2F links type 2 immune responses to plasmalogen-derived lysophospholipid production and IL-33 induction in keratinocytes

**Figure 1:**
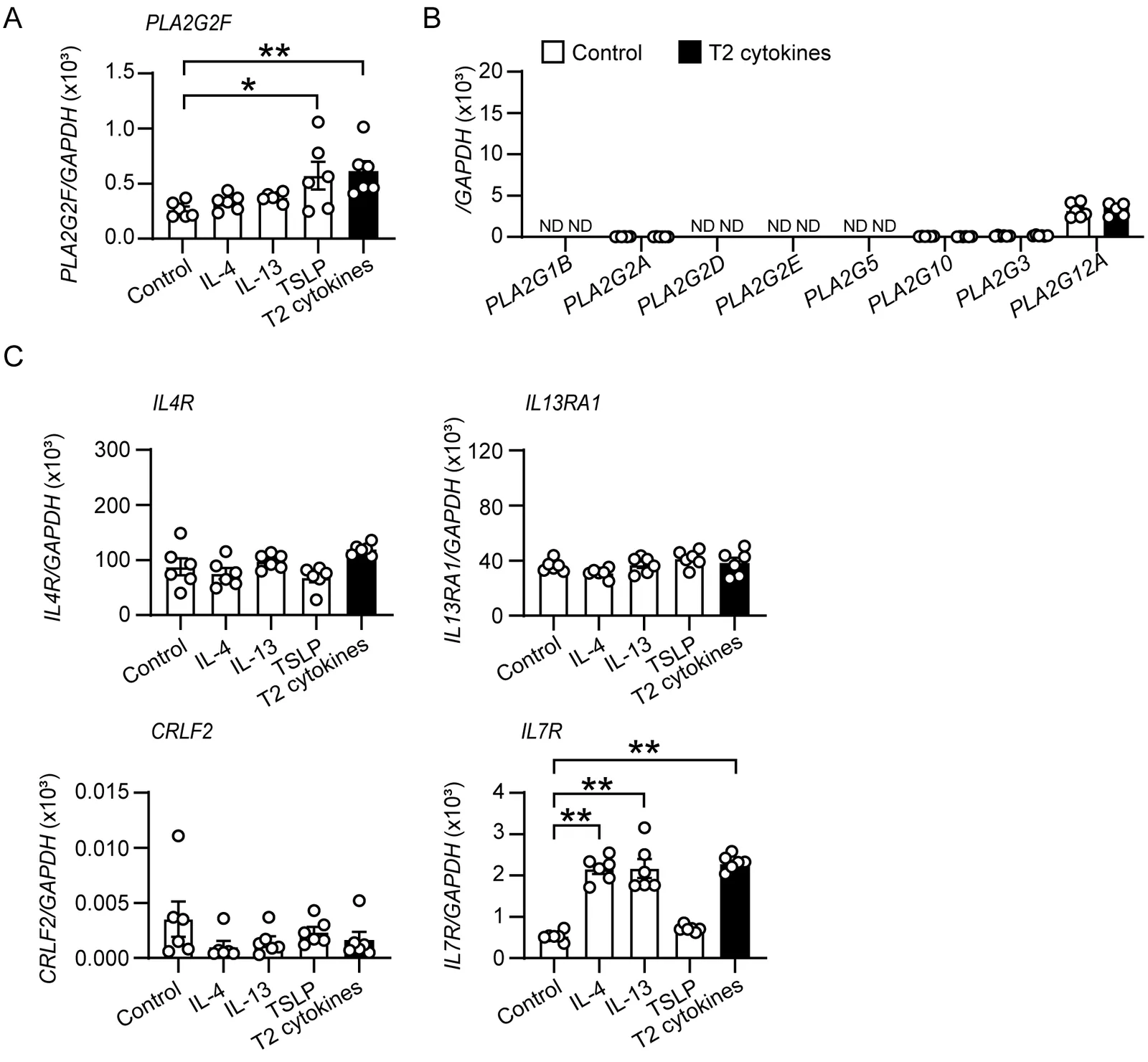
Type 2 cytokine-dependent induction of PLA2G2F and expression of cytokine receptors in NHEK/SVTERT3–5 cells. **(A)** NHEK/SVTERT3–5 cells were stimulated with IL-4, IL-13, TSLP, or a cocktail of type 2 (T2) cytokines (IL-4, IL-13, and TSLP), each at 10 ng/mL, for 24 h. *PLA2G2F* mRNA expression was quantified by qPCR and normalized to *GAPDH* (n = 6). **(B)** qPCR of sPLA_2_ isoforms in control and T2 cytokine-stimulated cells, normalized to *GAPDH* (n = 5). ND, not detected. **(C)** qPCR of *IL4R*, *IL13RA1*, *CRLF2*, and *IL7R*, normalized to *GAPDH* (n = 6). Data are presented as mean ± SEM. Statistical analyses were performed using two-way ANOVA by a multiple comparisons test **(A, C)** and unpaired two-tailed Student’s *t*-test **(B)**, *P* < 0.05 (*), *P* < 0.01 (**).

To investigate the role of PLA2G2F in keratinocyte responses under type 2 inflammatory conditions, we first examined its expression in primary newborn human epidermal keratinocytes (NHEK/SVTERT3-5) stimulate with IL-4, IL-13, TSLP, or a cocktail of type 2 cytokines (IL-4, IL-13, and TSLP). *PLA2G2F* mRNA expression showed a modestly increasing trend following stimulation with IL-4 and IL-13 and was significantly increased by TSLP and cytokine cocktail (hereafter Th2 conditions) (**Figure 1A**). Among the sPLA_2_ isoforms examined, only PLA2G2F was significantly induced by T2 cytokines, whereas the other isoforms were undetectable or minimally expressed, or unchanged, with the exception of *PLA2G12A*, which was consistently expressed in these cells (**Figure 1B**), suggesting dominant upregulation of PLA2G2F in keratinocytes under Th2 conditions. To evaluate the responsiveness of keratinocytes to type 2 cytokines, we next examined the expression of their corresponding receptors. Besides *IL4R* and *IL13RA1* that were constitutively expressed, *IL7R* (a subunit of heterodimetic TSLP receptor) was markedly induced by IL-4, IL-13, and the cytokine cocktail, although *CRLF2* (another subunit of TSLP receptor) remained low and was not significantly altered by cytokine stimulation (**Figure 1C**). These findings demonstrate that NHEK/SVTERT3-5 cells express several receptors for type 2 cytokines, that *IL7R* expression is enhanced by IL-4 or IL-13 under type 2 inflammatory conditions, and that TSLP induces *PLA2G2F* expression.

**Figure 2:**
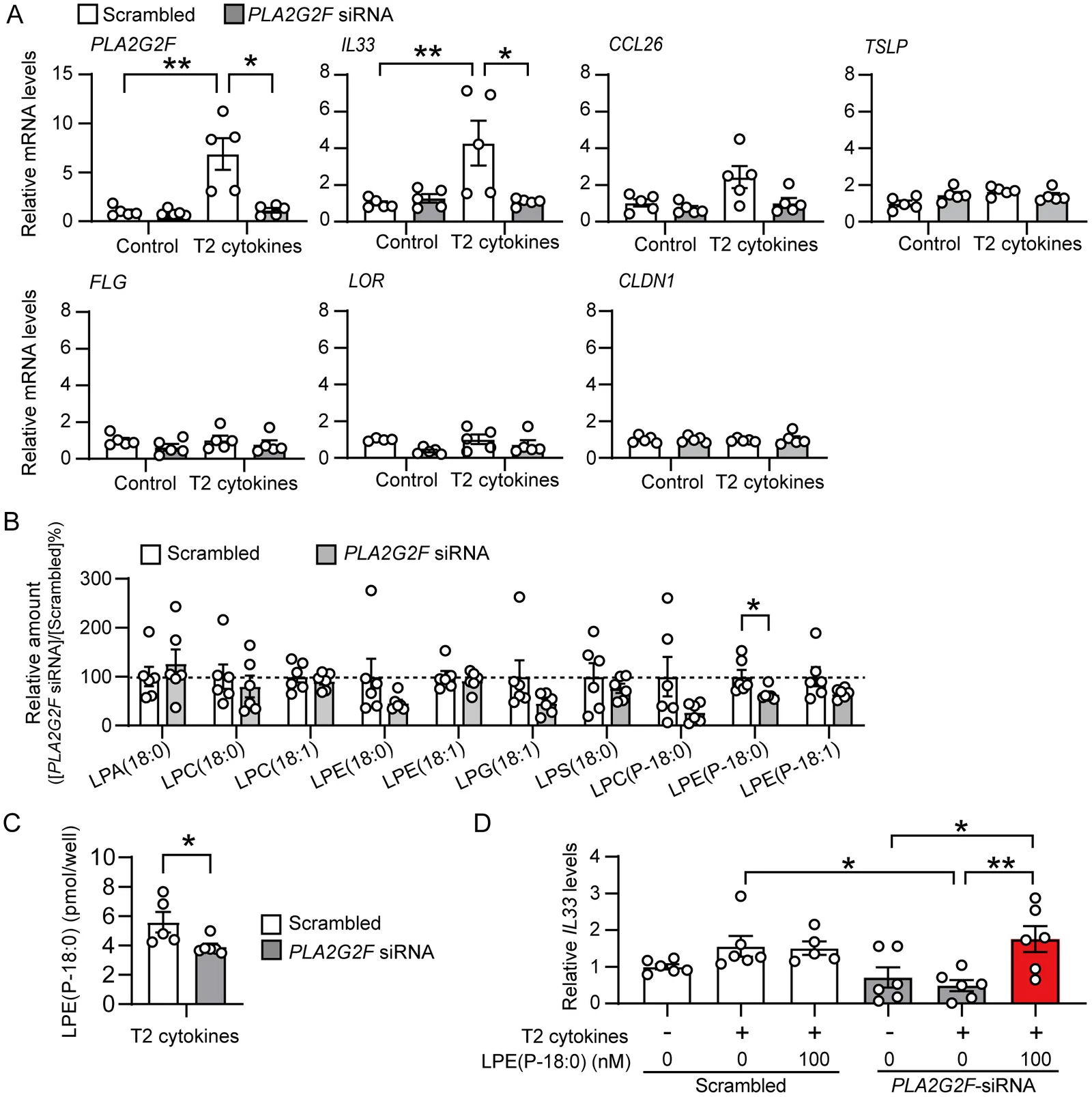
PLA2G2F regulates IL-33 expression through P-LPE production in NHEK/SVTERT3–5 cells under type 2 inflammatory conditions. **(A)** NHEK/SVTERT3–5 cells transfected with scrambled control or *PLA2G2F* siRNA were stimulated with a cocktail of T2 cytokines for 24 h. Relative mRNA expression of *PLA2G2F, IL33, CCL26, TSLP, FLG, LOR*, and *CLDN1* was quantified by qPCR and normalized to *GAPDH*. Data are presented relative to the scrambled control without T2 cytokine stimulation, which was set to 1 (n = 5). **(B)** Lipidomic analysis of lysophospholipid species in the culture supernatant of NHEK/SVTERT3–5 cells transfected with scrambled control or *PLA2G2F* siRNA and stimulated with a cocktail of T2 cytokines. Lipid levels are shown as percentages relative to scrambled control (dotted line = 100%) (n = 6). **(C)** Absolute quantification of LPE(P-18:0) in the culture supernatant of NHEK/SVTERT3–5 cells transfected with scrambled control or *PLA2G2F* siRNA and stimulated with or without the T2 cytokine cocktail (n = 6). **(D)** NHEK/SVTERT3–5 cells transfected with scrambled control or *PLA2G2F* siRNA were stimulated with T2 cytokines for 24 h in the presence or absence of LPE(P-18:0) (100 nM), as indicated. Relative *IL33* mRNA expression was determined by qPCR and normalized to *GAPDH* (n = 6). Data are presented as mean ± SEM. Statistical analyses were performed using two-way ANOVA by a multiple comparisons **(A, D)** test and unpaired two-tailed Student’s *t*-test **(B, C)**, *P* < 0.05 (*), *P* < 0.01 (**).

To assess the downstream effects of PLA2G2F in keratinocytes, we performed siRNA-mediated knockdown, which successfully abrogated type 2 cytokine-induced *PLA2G2F* expression (**Figure 2A**). *PLA2G2F* knockdown significantly suppressed *IL33* expression, whereas *CCL26* (eotaxin-3) showed a tendency toward reduced expression without reaching statistical significance. In contrast, the expression of keratinocyte differentiation markers (*FLG*, *LOR*, and *CLDN1*) as well as *TSLP* was unchanged (**Figure 2A**). These results suggest that PLA2G2F is induced in human keratinocytes under type 2 cytokine conditions and contributes to the amplification of the epithelial alarmin response by selectively upregulating IL-33. We previously demonstrated that PLA2G2F preferentially mobilizes plasmalogen-type lysophospholipid P-LPE in the skin of patients with Th17-driven psoriasis (32). To explore the lipid metabolism driven by PLA2G2F under type 2 cytokine conditions, we performed lipidomic analysis of lysophospholipids in the culture supernatant of NHEK/SVTERT3–5 cells following *PLA2G2F* knockdown. Among the lysophospholipid species analyzed thus far, P-LPE with stearic acid (LPE(P-18:0)) was significantly reduced following *PLA2G2F* knockdown, whereas most other lysophospholipid species showed no statistically significant changes, although LPE(18:0), LPC(P-18:0), and LPE(P-18:1) also exhibited a tendency to decrease (**Figure 2B**). To validate the lipidomic findings, we next performed absolute quantification of extracellular LPE(P-18:0). Consistent with the lipidomic analysis, *PLA2G2F* knockdown significantly reduced extracellular LPE(P-18:0) levels under type 2 cytokine conditions (**Figure 2C**). To determine whether reduced LPE(P-18:0) production contributes to the suppression of IL-33 expression following *PLA2G2F* knockdown, exogenous LPE(P-18:0) was added to *PLA2G2F*-silenced NHEK/SVTERT3–5 cells. LPE(P-18:0) restored *IL-33* expression in *PLA2G2F*-knockdown cells (**Figure 2D**), suggesting that PLA2G2F regulates IL-33 expression through LPE(P-18:0). Collectively, these findings suggest that PLA2G2F is induced under type 2 conditions, preferentially mobilizes P-LPE, and promotes an IL-33-mediated epithelial response in human keratinocytes.

### PLA2G2F deficiency attenuates type 2 inflammation and skin pathology in an atopic dermatitis model

**Figure 3:**
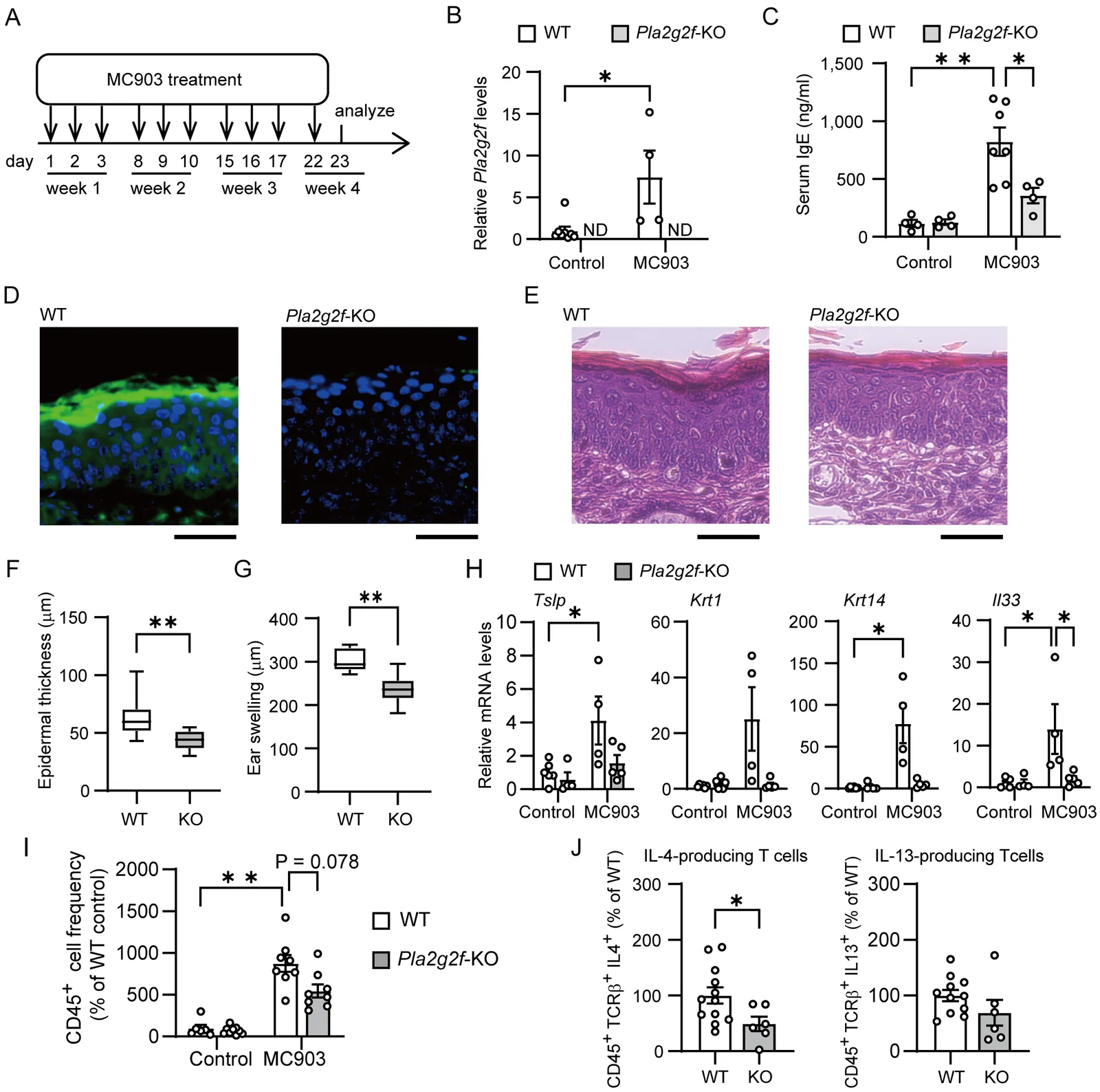
PLA2G2F deficiency attenuates MC903-induced atopic dermatitis–like inflammation *in vivo.* **(A)** Experimental scheme of the MC903-induced atopic dermatitis–like model. **(B)** qPCR of *Pla2g2f* in the skin of WT and *Pla2g2f*-KO mice with or without MC903 treatment for 4 weeks, with expression (normalized to *Gapdh*) in vehicle (control)-treated WT mice as 1 (n = 4 - 8). ND, not detected. **(C)** Serum IgE levels in WT and *Pla2g2f*-KO mice under control and MC903-treated conditions (n = 4 - 7). **(D)** Representative immunofluorescence staining of PLA2G2F (green), followed by counterstaining of nuclei with DAPI (blue), in the epidermis of MC903-treated WT and *Pla2g2f*-KO mice. Scale bar, 50 μm. **(E)** Representative histological images of ear skin sections from MC903-treated WT and *Pla2g2f*-KO mice stained with hematoxylin and eosin. Scale bar, 50 μm. **(F, G)** Quantification of epidermal **(F)** and ear **(G)** thickness in WT and *Pla2g2f*-KO mice following MC903 treatment, as determined from the histological images in **(E)** (n = 12 - 19). **(H)** Relative expression levels of indicated genes (*Tslp*, *Krt1*, *Krt14*, and *Il33*) normalized to *Gapdh* in skin tissues from WT and *Pla2g2f*-KO mice under control and MC903-treated conditions, with expression in control WT mice as 1 (n = 5 - 7). **(I)** Flow cytometric analysis of CD45^+^ immune cells in skin tissues of WT and *Pla2g2f*-KO mice under control and MC903-treated conditions (n = 6 - 9). **(J)** Flow cytometric analysis of IL-4– or IL-13–producing CD45^+^ TCRβ^+^ T cells in skin tissues of MC903-treated WT and *Pla2g2f*-KO mice (n = 6 - 12). Data are presented as mean ± SEM **(B, C, H, I, J)** or box-and-whisker plot (F, G). Statistical analysis was performed using two-way ANOVA with Sidak’s multiple comparisons test **(B, C, H, I)** or unpaired two-tailed Student’s *t*-test **(F, G, J)**, *P* < 0.05 (*), *P* < 0.01 (**). Representative flow cytometric gating strategies are shown in **Supplementary Figures 1**, **2**.

To determine whether the *in vitro* findings could be translated into pathophysiology *in vivo*, we examined the role of PLA2G2F in a mouse model of atopic dermatitis induced by the topical application of MC903, an agent that upregulates TSLP and thereby elicits type 2 inflammation (36) (**Figure 3A**). Consistent with our *in vitro* observations (**Figures 1**, **2**), *Pla2g2f* expression was markedly induced in the skin following MC903 treatment in wild-type (WT), but not in *Pla2g2f*-deficient (KO), mice (**Figure 3B**). MC903-treated WT mice showed elevated serum IgE levels, whereas this response was significantly reduced in *Pla2g2f*-KO mice (**Figure 3C**). Immunostaining for PLA2G2F revealed that its positive signal was predominantly located in the uppermost region of the epidermis, likely the stratus corneum, of MC903-treated WT, but not KO, skin (**Figure 3D**). Histological analysis revealed that MC903-induced epidermal hyperplasia was markedly suppressed in *Pla2g2f*-KO mice (**Figures 3E, F**). Additionally, ear swelling was significantly attenuated in KO mice compared with WT controls (**Figure 3G**), indicating reduced inflammatory responses. Consistently, the cutaneous expression of type 2 inflammation-associated genes, including *Tslp* and *Il33*, was decreased in KO mice, and keratinocyte differentiation markers (*Krt1* and *Krt14*) exhibited a similar trend (**Figure 3H**). Flow cytometric analysis revealed that the increased proportion of CD45^+^ immune cells in the skin of WT mice following MC903 treatment was less pronounced in *Pla2g2f*-KO mice (**Figure 3I**; **Supplementary Figure 1**). Among the immune cell populations, the proportion of IL-4–producing CD4^+^ T cells was significantly reduced, and that of IL-13–producing T cells showed a decreasing trend in MC903-treated *Pla2g2f*-KO skin (**Figure 3J**; **Supplementary Figure 2**). Taken together, these results suggest that PLA2G2F participates in the development of type 2 inflammation and epidermal pathology *in vivo*.

### PLA2G2F promotes plasmalogen-derived lysophospholipid accumulation in the stratum corneum during dermatitis

**Figure 4:**
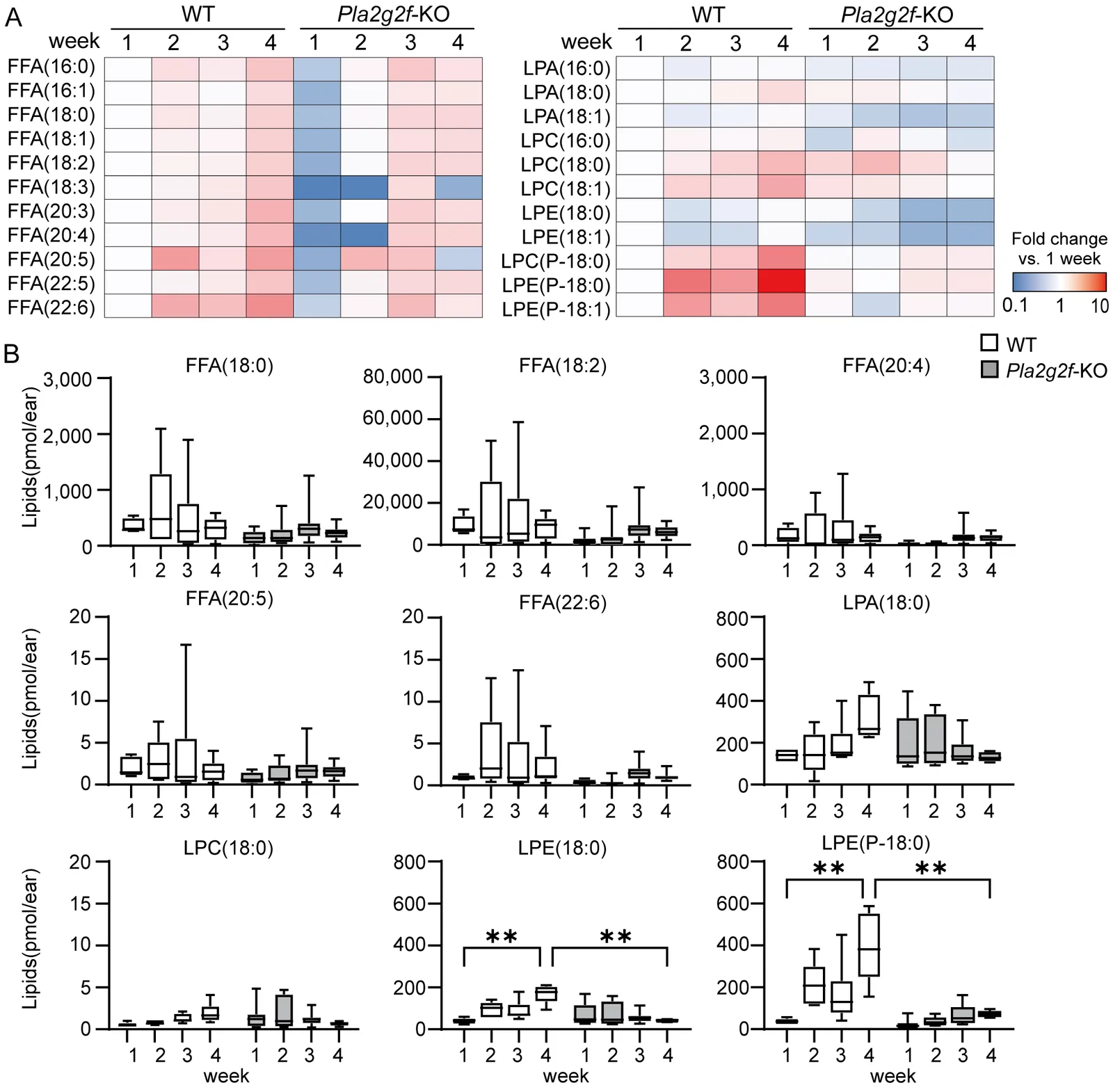
PLA2G2F drives plasmalogen-derived lysophospholipid production in the stratum corneum during atopic dermatitis-like inflammation. **(A)** Heatmap showing fold changes of lipid species in the stratum corneum from ear tissues of WT and *Pla2g2f*-KO mice after MC903 treatment for the indicated periods (weeks 1–4), with their levels in WT mice at week 1 as 1. **(B)** Quantified values of selected lipid species in **(A)**. Data are presented as box-and-whisker plots (n = 5–9 mice per group). Statistical analysis was performed using two-way ANOVA followed by appropriate multiple comparison test. *P* < 0.01 (**).

Having established that PLA2G2F contributes to Th2-driven inflammatory responses *in vivo*, we sought to gain insights into the underlying mechanisms in the epidermis. Given that PLA2G2F is predominantly localized in the uppermost epidermis (**Figure 3D**) (32), we focused on the lipid composition of this epidermal compartment. Stratum corneum samples were collected non-invasively using a swab-based method, which enabled repeated sampling during disease progression. Lipidomic analyses were performed using samples obtained from WT and *Pla2g2f*-KO mice subjected to MC903-induced dermatitis. Heatmap analysis revealed dynamic changes in lipid profiles over time in WT mice, including a marked increase in P-LPE species, whereas these changes were substantially attenuated in *Pla2g2f-*KO mice (**Figure 4A**). Quantitative analysis further demonstrated that LPE(P-18:0) levels in the stratum corneum were significantly elevated in WT mice in a time-dependent manner following MC903 treatment, whereas this induction was markedly suppressed in *Pla2g2f*-KO mice (**Figure 4B**). Although LPE(18:0) also exhibited a modest yet significant increase following MC903 treatment and was reduced in *Pla2g2f*-KO mice, the absolute abundance of LPE(P-18:0) was approximately 2–3-fold greater than that of LPE(18:0), suggesting that plasmalogen-derived lysophospholipids constitute the major fraction influenced by PLA2G2F (**Figure 4B**). Other lipid classes, including free fatty acids (FFA), LPA and LPC, showed no consistent genotype-dependent changes, although some tended to be modestly lower in *Pla2g2f*-KO mice. These findings suggest that PLA2G2F preferentially generates plasmalogen-derived lysophospholipids in the stratum corneum and highlight the usefulness of non-invasive swab-based sampling for monitoring lipid dynamics during skin inflammation.

### PLA2G2F directly generates plasmalogen-derived lysophospholipids from skin lipid substrates

**Figure 5:**
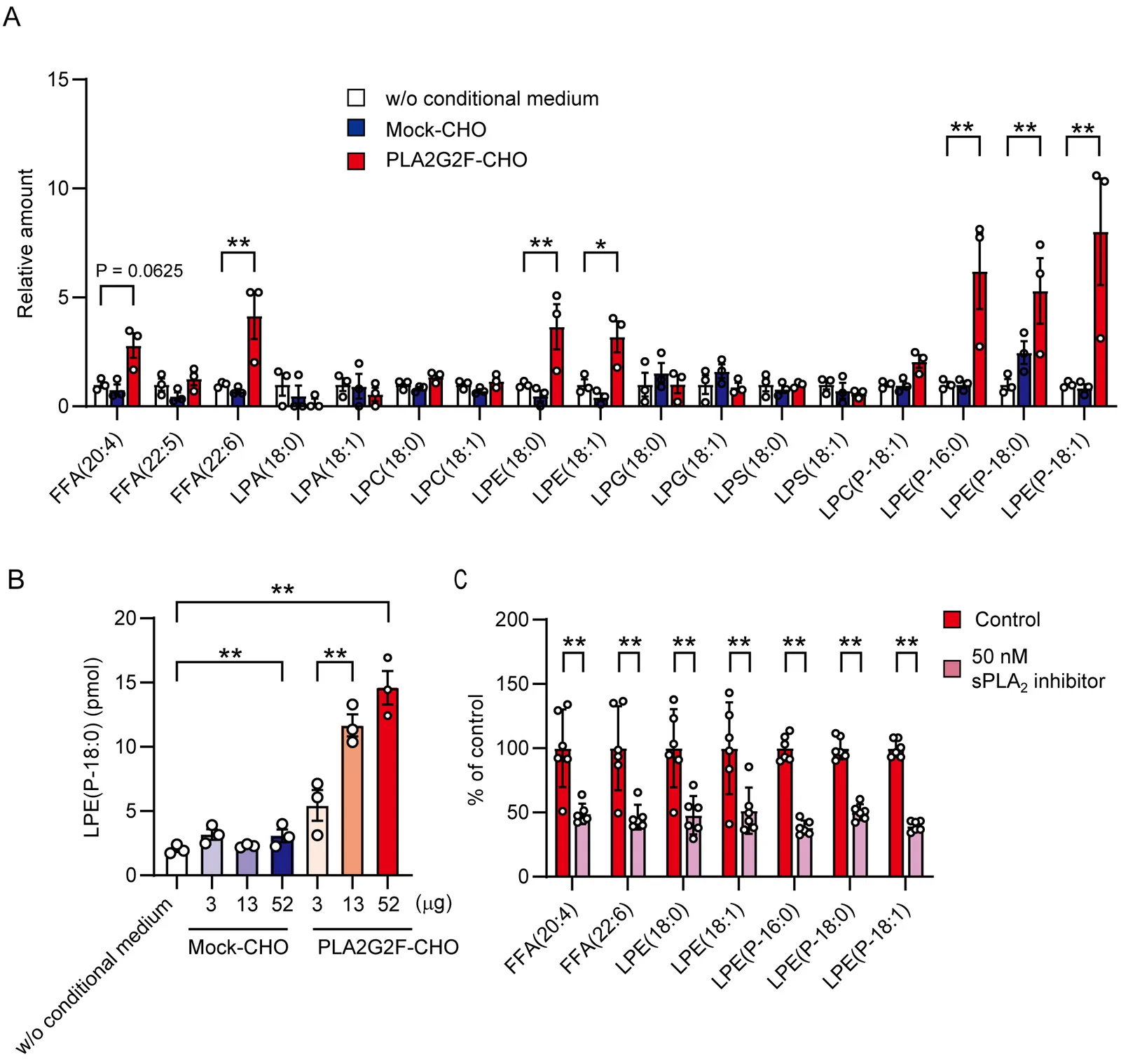
PLA2G2F-dependent production of lysophospholipids and fatty acids from skin-extracted phospholipids and its inhibition by an sPLA_2_ inhibitor. **(A)** Lipidomic analysis of lipids extracted from ear skin of MC903-treated mice that were incubated for 30 min with conditioned medium from control (Mock-CHO) or PLA2G2F-expressing (PLA2G2F-CHO) cells (n = 3). **(B)** Quantification of LPE(P-18:0) production after incubation with increasing amounts of conditioned medium from Mock-CHO and PLA2G2F-CHO cells (n = 3). **(C)** Effects of the sPLA_2_ inhibitor 12e (2-(3-(2-amino-2-oxoacetyl)-1-benzyl-2-ethyl-1H-6,7-benzoindol-4-yloxy) acetic acid) on production of the indicated lipids, with their levels in the absence of the inhibitor (control) as 100% (n = 6). Data are presented as mean ± SEM. Statistical analysis was performed using two-way ANOVA with Tukey’s multiple comparisons test **(A, B)** or unpaired two-tailed Student’s *t*-test **(C)**, *P* < 0.05 (*), *P* < 0.01 (**).

To directly confirm that PLA2G2F enzymatically generated plasmalogen-derived lysophospholipids from skin lipids, we performed an *in vitro* enzymatic reaction using total phospholipid extracts from MC903-treated mouse skin as a substrate. These lipid extracts were incubated with conditioned medium collected from control (Mock-CHO) or PLA2G2F-expressing CHO cells, followed by lipidomic analysis. Incubation with the PLA2G2F-containing supernatant obtained from PLA2G2F-expressing CHO cells markedly increased the levels of P-LPE species including LPE(P-16:0), LPE(P-18:0) and LPE(P-18:1) and to a lesser extent those of LPE species including LPE(18:0) and LPE(18:1), as well as those of polyunsaturated fatty acids including arachidonic acid (20:4) and docosahexaenoic acid (22:6), compared to the supernatant from Mock-CHO cells or no enzyme control (**Figure 5A**). Consistently, quantitative analysis demonstrated a dose-dependent increase in LPE(P-18:0) production in response to increasing amounts of PLA2G2F-containing supernatant, whereas the Mock-CHO supernatant showed a minimal effect (**Figure 5B**). Inhibition of sPLA_2_ activity by 2-(3-(2-amino-2-oxoacetyl)-1-benzyl-2-ethyl-1H-6,7-benzoindol-4-yloxy) acetic acid, a varespladib derivative that is capable of potently inhibiting PLA2G2F [corresponding to compound 12e in Oslund et al. (38)], significantly reduced the generation of P-LPE and FFA species (**Figure 5C**), indicating that the observed changes in lipid profiles are mediated by the enzymatic activity of PLA2G2F. Together, these results confirm that PLA2G2F preferentially hydrolyzes plasmalogen with *sn*-2 polyunsaturated fatty acids to produce P-LPE species from skin phospholipids, providing mechanistic evidence that links PLA2G2F activity to lipid changes observed *in vitro* and *in vivo*.

### PLA2G2F-dependent plasmalogen metabolism regulates itch responses in atopic dermatitis

**Figure 6:**
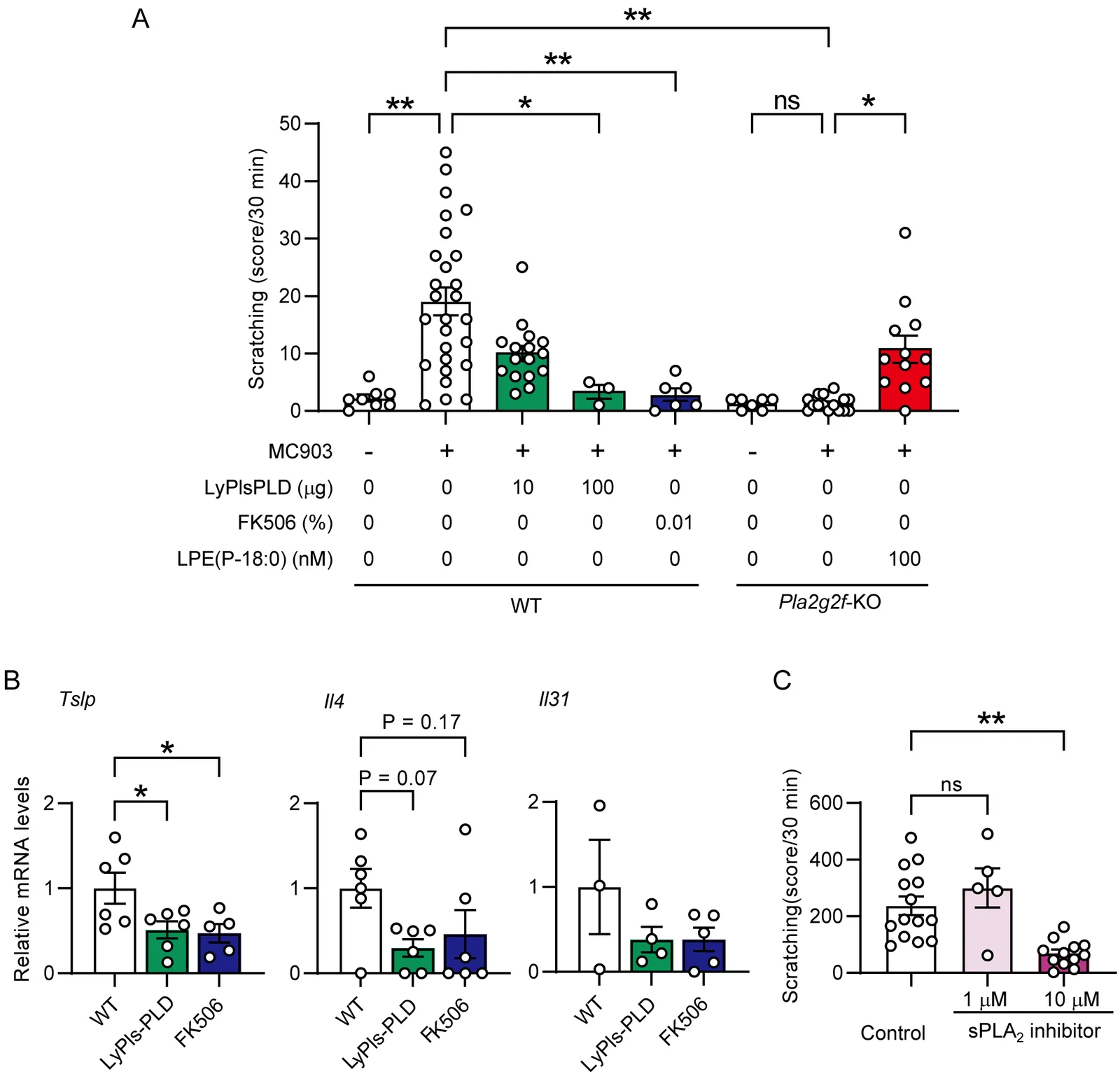
PLA2G2F-dependent P-LPE signaling regulates itch in MC903-induced dermatitis. **(A)** Scratching behavior (number of scratching bouts per 30 min) in WT and *Pla2g2f* -KO mice subjected to MC903-induced dermatitis. Mice were topically treated with LyPls-PLD, FK506, or LPE(P-18:0) on day 22 (**Figure 3A**) at the indicated doses, followed by analysis on day 23 (n = 3 - 27). **(B)** qPCR of inflammatory and itch-related genes (*Tslp, Il4*, and *Il31*) normalized to *Gapdh* in the skin of MC903-challenged WT mice following treatment with LyPls-PLD or FK506, with expression without drug treatment as 1 (n = 3 - 5). **(C)** Effects of an sPLA_2_ inhibitor on scratching behavior in MC903-treated WT mice. Scratching response was quantified following treatment with the indicated doses of the inhibitor (n = 5 - 12). Data are presented as mean ± SEM. Statistical analysis was performed using one-way or two-way ANOVA followed by appropriate multiple comparison tests. *P* < 0.05 (*), *P* < 0.01 (**); ns, not significant.

Itch, or pruritus, is defined as an unpleasant feeling on the skin that triggers an urge to scratch. Under chronic conditions such as atopic dermatitis, this natural reflex spirals into an unbearable “itch-scratch-itch” cycle, exacerbating skin lesions that present a significant clinical challenge. We found that MC903-treated WT mice exhibited a marked increase in scratching behavior, whereas this response was markedly attenuated in *Pla2g2f*-KO mice (**Figure 6A**), implying that PLA2G2F contributes to the itch response during atopic dermatitis. To examine the contribution of plasmalogen-derived lysophospholipids to itching, we pharmacologically modulated their cutaneous levels. Topical treatment with recombinant *Actinomycete* LyPls-PLD, which specifically degrades P-LPE (33, 37), significantly reduced scratching behavior in WT mice. Similarly, treatment with FK506, an immunosuppressant, suppressed the MC903-induced itch response (**Figure 6A**), supporting the involvement of inflammation. Indeed, the expression of the epithelial alarmin *Tslp* was significantly reduced following LyPls-PLD or FK506 treatment, and *Il4* and *Il31* also showed a tendency toward deceased expression (**Figure 6B**). Conversely, the topical administration of LPE(P-18:0) partially restored scratching behavior in *Pla2g2f*-KO mice (**Figure 6A**). Furthermore, pharmacological inhibition of sPLA_2_ activity by the varespladib derivative 12e in MC903-treated mice dampened scratching behavior (**Figure 6C**). Collectively, these findings suggest that PLA2G2F-driven plasmalogen metabolism is functionally coupled with the itch response and that pharmacological targeting of this lipid pathway can efficiently alleviate pruritus in atopic dermatitis.

### PLA2G2F deficiency decreases the expression of IL-4 and IL-31 receptors in DRG neurons

**Figure 7:**
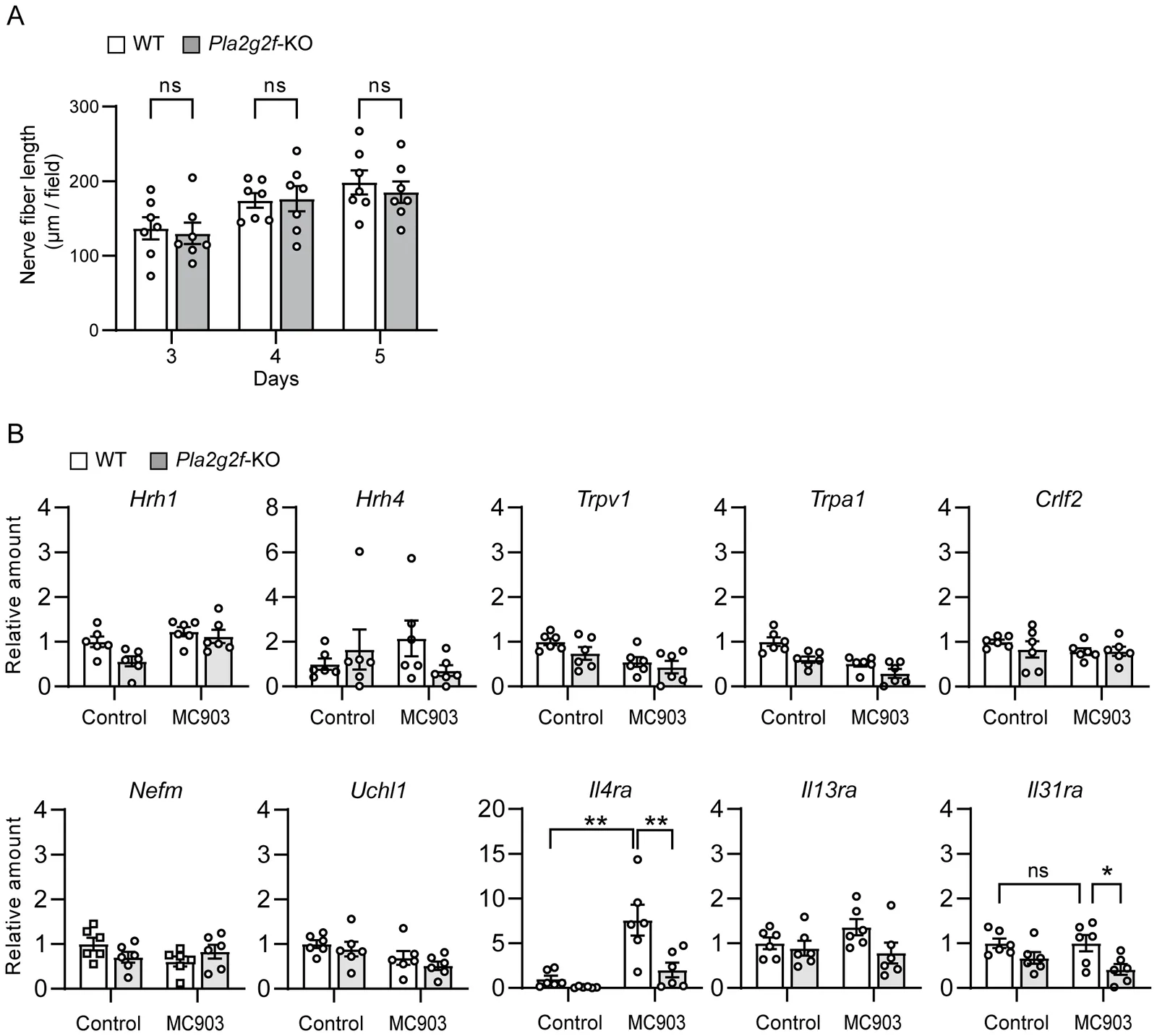
PLA2G2F deficiency alters the expression of IL-4 and IL-31 receptors in DRG neurons. **(A)** Quantification of neurite outgrowth from DRG neurons isolated from WT and *Pla2g2f-*KO mice. DRG neurons were cultured for the indicated time periods, and total neurite length per field was measured (n = 7). **(B)** qPCR of neuronal and itch-related genes in DRG tissues isolated from WT and *Pla2g2f-*KO mice with vehicle (control) or MC903 treatment, with expression in control WT mice as 1 (n = 6). Data are presented as mean ± SEM. Statistical analysis was performed using two-way ANOVA with Tukey’s multiple comparison test. *P* < 0.05 (*), *P* < 0.01 (**); ns, not significant.

The itch-sensing neurons that innervate the skin extend their peripheral nerve endings into the epidermis and dermis, whereas their cell bodies reside in the dorsal root ganglia (DRG). To investigate whether PLA2G2F promotes pruritus by influencing neuronal structure or function, DRG were isolated, dissociated, and cultured *in vitro*, and neurite outgrowth was quantified. No significant difference in neurite length was observed between the WT and *Pla2g2f*-KO neurons (**Figure 7A**). qPCR of DRG neurons revealed that the expression levels of itch-related receptors, including histamine receptors (*Hrh1* and *Hrh4*), transient receptor potential (TRP) channels (*Trpv1* and *Trpa1*), and TSLP and IL-13 receptors (*Crlf2* and *Il13ra*, respectively), as well as those of neuronal markers (*Nefm* and *Uchl1*), were comparable between WT and *Pla2g2f*-KO mice (**Figure 7B**). Notably, MC903-induced upregulation of IL-4 receptor (*Il4ra*) and constitutive expression of IL-31 receptor (*Il31ra*) were significantly reduced in *Pla2g2f*-KO mice relative to WT mice (**Figure 7B**). Collectively, these results suggest that the reduced itch response observed in *Pla2g2f*-KO mice is unlikely to be due to alterations in neuronal structure or intrinsic neuronal signaling and instead supports a role for PLA2G2F-mediated epithelial–immune crosstalk, potentially involving altered IL-4 and IL-31 responsiveness, in itch regulation.

### Plasmalogen-derived lysophospholipids are elevated in human atopic dermatitis and associate with disease severity

**Figure 8:**
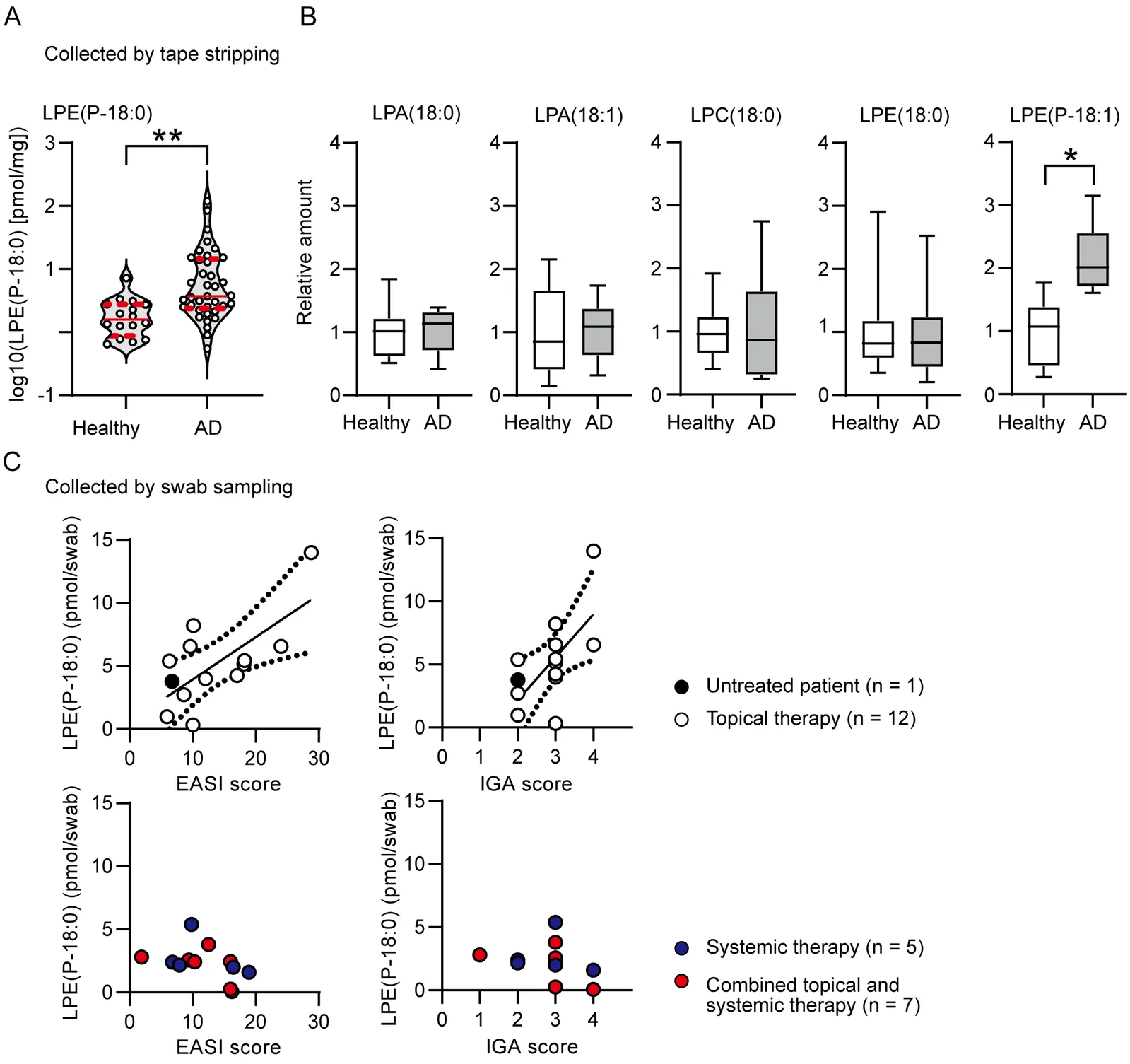
Correlation analysis of P-LPE levels and disease severity in patients with atopic dermatitis. **(A)** Quantification of LPE(P-18:0) levels in stratum corneum samples collected by tape stripping from healthy individuals (n = 16) and patients with atopic dermatitis (AD) (n = 37). Samples in healthy control were identical to those reported in our previous study (33). All samples were analyzed simultaneously under identical experimental conditions to minimize batch effects. **(B)** Relative amounts of indicated lipid species in stratum corneum samples from healthy individuals (n = 10) and AD patients (n = 8), with levels in healthy control as 1. **(C)** Correlation of LPE(P-18:0) levels with disease severity. Stratum corneum samples were collected by swab sampling, and LPE(P-18:0) levels were plotted against EASI or IGA scores. Open, closed, blue and red circles indicate patients receiving topical therapy with corticosteroids (n = 12), an untreated patient (n = 1), patients receiving systemic therapy with dupilmab (n = 5), and patients receiving combined topical and systemic therapy (n = 7), respectively. The data are presented as violin **(A)** or box-and-whisker **(B)** plots. Statistical analysis was performed using the Mann–Whitney U test **(A, B)** and correlation analysis **(C)**. *P* < 0.05 (*), *P* < 0.01 (**).

To assess the clinical relevance of our findings, we examined whether plasmalogen-derived lysophospholipids were altered in patients with atopic dermatitis. Stratum corneum samples were collected from patients with atopic dermatitis and control (healthy) subjects by tape-stripping, and their lipid levels were quantified by LC–MS/MS. The levels of LPE(P-18:0) were significantly elevated in patients with atopic dermatitis compared to healthy controls (**Figure 8A**). Another plasmalogen-derived species, LPE(P-18:1), also increased in patients, whereas most other lysophospholipids showed no significant changes (**Figure 8B**), indicating the selective elevation of plasmalogen-derived lysophospholipids under disease conditions in humans. Next, we evaluated the relationship between LPE(P-18:0) levels and disease severity using an independent cohort in which stratum corneum samples were collected non-invasively using a swab-based method. Correlation analyses revealed a positive association between LPE(P-18:0) levels and clinical severity scores, including the Eczema Area and Severity Index (EASI) and the Investigator’s Global Assessment (IGA), in patients receiving topical therapy (*e.g*., corticosteroids) (**Figure 8C**; **Tables 1**, **2**). In contrast, this association was not observed in patients treated with dupilumab, a monoclonal antibody targeting IL-4/IL-13 signaling (41, 42). These results suggest that the association between plasmalogen-derived lysophospholipids and disease activity may be modulated by therapeutic inhibition of IL-4/IL-13 signaling.

**Table 1:** Correlation analysis between LPE(P-18:0) levels and EASI score.

EASI score				
Treatment	Pearson correlation		Spearman correlation	
Untreated	–	–	–	–
Topical	r = 0.6783	P = 0.0153	r = 0.5175	P = 0.0888
Systemic	r = -0.3935	P = 0.5122	r = -0.7000	P = 0.2333
Topical + Systemic	r = -0.5302	P = 0.2208	r = -0.6847	P = 0.0984

The Pearson and Spearman correlation coefficients (r) and corresponding P values are shown for each treatment group (untreated, topical therapy, systemic therapy, and combined topical and systemic therapy). Correlations were calculated between LPE(P-18:0) levels (pmol/swab) and EASI scores.

**Table 2:** Correlation analysis between LPE(P-18:0) levels and IGA score.

IGA score				
Treatment	Pearson correlation		Spearman correlation	
Untreated	–	–	–	–
Topical	r = 0.6383	P = 0.0255	r = 0.5195	P = 0.0525
Systemic	r = -0.0528	P = 0.9328	r = -0.5270	P = 0.4667
Topical + Systemic	r = -0.4701	P = 0.2871	r = -0.6682	P = 0.1429

The Pearson and Spearman correlation coefficients (r) and corresponding P values are shown for each treatment group (untreated, topical therapy, systemic therapy, and combined topical and systemic therapy). Correlations were calculated between LPE(P-18:0) levels (pmol/swab) and IGA scores.

## Discussion

Atopic dermatitis is characterized by type 2 inflammation and severe itching (1); however, the molecular mechanisms linking epidermal lipid metabolism to immune signaling remain incompletely understood. Although the eicosanoid (PG and LT) pathways have been well characterized in atopic dermatitis lesions (10), the contribution of lysophospholipids to disease pathogenesis remains largely unexplored. In the present study, we identified the PLA2G2F/P-LPE axis that connects keratinocyte-derived lipid signaling to type 2 immune responses and itching, potentially involving IL-33–associated alarmin signaling and IL-4-mediated neuronal activation. Importantly, genetic or pharmacological inhibition of the PLA2G2F/P-LPE axis efficiently alleviates the itch response associated with atopic dermatitis, highlighting this unique lipid pathway as a novel drug target for pruritus. Moreover, P-LPE in the stratum corneum collected using non-invasive methods may represent a novel diagnostic marker of atopic dermatitis.

Herein, we show that PLA2G2F is upregulated by type 2 cytokines, promotes the production of plasmalogen-derived P-LPE species, and amplifies alarmin expression, particularly that of IL-33 (43), in keratinocytes. Our findings support a model in which IL-4 and IL-13 enhance keratinocyte responsiveness to TSLP through induction of IL7R, TSLP induces PLA2G2F, PLA2G2F generates P-LPE, and P-LPE promotes IL-33 expression, suggesting that the PLA2G2F/P-LPE axis may serve as a molecular bridge linking multiple type 2 cytokine signals. Supporting this model, the significant reduction of LPE(P-18:0) following *PLA2G2F* knockdown supports a role for PLA2G2F in P-LPE production under type 2 inflammatory conditions. Our previous studies demonstrated that IL-22–induced PLA2G2F contributes to Th17-driven psoriasis by producing P-LPE (32, 33) and that this sPLA_2_ also participates in the exacerbation of Th1-driven contact dermatitis and skin tumor progression (32). Our findings extend this paradigm by demonstrating that the PLA2G2F/P-LPE axis is also involved in Th2-driven inflammation, indicating that PLA2G2F functions as a broad regulator of lipid-mediated cutaneous immune responses across multiple inflammatory contexts. The physiological relevance of this pathway was supported by *in vivo* experiments using an MC903-induced dermatitis model, in which the genetic ablation of *Pla2g2f* resulted in reduced epidermal hyperplasia, serum IgE levels, type 2 inflammation, and itch response, accompanied by reduced production of P-LPE in the stratum corneum. Remarkably, PLA2G2F-dependent lipid metabolism contributed to MC903-induced itch, as evidenced by reduced scratching and partial restoration by exogenous P-LPE in *Pla2g2f*-deficient mice, as well as its abrogation by the pharmacological degradation of P-LPE or inhibition of sPLA_2_ activity in WT mice, supporting a model in which P-LPE acts downstream of PLA2G2F to promote itch in atopic dermatitis. This effect was accompanied by reduced *Il4ra* expression in DRG neurons and decreased IL-4–producing T cells, suggesting that PLA2G2F primarily regulates itch through epithelial–immune interactions. Consistent with this, the association between P-LPE levels and disease severity was lost in patients treated with dupilumab, further supporting the hypothesis that this pathway depends on IL-4/IL-13 signaling.

Recent studies have shown that arachidonic acid–derived lipid mediators, including PGs and LTs, regulate type 2 immunity, barrier dysfunction, and pruritus in atopic dermatitis (10). In contrast, we identified a distinct lipid signaling pathway mediated by plasmalogen-derived P-LPE. Unlike conventional arachidonic acid metabolites that are primarily generated by intracellular cPLA_2_, P-LPE represents a structurally unique class of lipid mediators generated by sPLA_2_ in the extracellular milieu of the suprabasal epidermis. This view agrees with the recently emerging concept that sPLA_2_s drive extracellular lysophospholipid signaling by acting on extracellular vesicles (29, 44–46). In addition to their roles in immune regulation and pruritus, lipid mediators have been implicated in controlling skin barrier function. Arachidonic acid–derived lipid mediators modulate epidermal barrier integrity through their effects on keratinocyte differentiation and tight junction formation (17, 19). Our findings suggest that PLA2G2F-dependent P-LPE metabolism may represent an additional mechanism that influences barrier-related processes. Although our study primarily focused on inflammatory and itch responses, the impaired epidermal differentiation observed in *Pla2g2f*-deficient mice (32) suggests that P-LPE–mediated signaling contributes to the regulation of keratinocyte differentiation. Given that type 2 cytokines such as IL-4 and IL-13 disrupt barrier integrity (1–3), the interplay between the PLA2G2F/P-LPE axis and type 2 cytokine signaling may provide an additional link between lipid metabolism and barrier dysfunction in atopic dermatitis. While the direct effects of P-LPE on barrier components remain to be determined, this pathway may contribute indirectly to proper skin barrier function by modulating epithelial–immune interactions.

Current therapeutic strategies for pruritus in atopic dermatitis include antihistamines, corticosteroids, calcineurin inhibitors, JAK inhibitors and biologics targeting type 2 cytokines such as IL-4, IL-13 and IL-31 (1–3). While these treatments are effective in ameliorating inflammation and itch in many patients, a substantial proportion of patients exhibit incomplete responses or persistent pruritus (47, 48), implying the presence of additional itch-promoting pathways that remain to be elucidated. Notably, antihistamines are often ineffective in atopic dermatitis, highlighting the importance of non-histaminergic mechanisms of itch (47). In this context, our findings provide evidence that the PLA2G2F/P-LPE axis acts as a previously unrecognized lipid-mediated pathway that controls itch. Unlike cytokine-targeting biologics, which incur high medical costs and can cause unfavorable side effects due to the suppression of the systemic immune system, targeting PLA2G2F, an epidermal-specific sPLA_2_, by topical application of specific inhibitors may offer an alternative strategy to avoid undesirable complications and complement existing therapies, particularly for patients with residual itch despite adequate control of inflammation. Although the sPLA_2_ inhibitor (12e) has been reported to potently inhibit PLA2G2F (38), it also inhibits other sPLA_2_ isoforms. However, PLA2G2F is the predominant sPLA_2_ expressed in primary keratinocytes (49). Although PLA2G10 had been reported to be expressed in transformed human keratinocytes (50), our subsequent studies provided compelling evidence that PLA2G10 is expressed at much lower levels and is predominantly localized to the outer root sheath of hair follicles rather than the epidermis in mouse skin (51). Consistent with these observations, PLA2G2F was the only sPLA_2_ induced under Th2 conditions in our keratinocyte model, supporting the notion that inhibition of PLA2G2F largely accounts for the effects of sPLA_2_ inhibitor in the present study. Furthermore, the comparable phenotypes observed in *Pla2g2f*-deficient mice further support PLA2G2F as the principal pharmacological target responsible for the effects of sPLA_2_ inhibitor in this model.

Plasmalogen metabolism is dynamically regulated by both biosynthetic and degradative pathways; however, its functional output remains unclear. Plasmalogens are a distinct subclass of glycerophospholipids characterized by a vinyl ether linkage at the *sn-1* position, conferring unique structural, signaling, and antioxidant properties (52, 53). Although their biosynthesis has been well characterized, recent studies have identified the lysoplasmalogenases TMEM86A and TMEM86B as key regulators of plasmalogen catabolism (54, 55). Genetic ablation of these enzymes leads to the accumulation of various plasmalogen and lysoplasmalogen species, including LPE(P-18:0), in several tissues, including the liver and adipose tissue. Notably, TMEM86A deficiency in adipocytes enhanced protein kinase A signaling by inhibiting phosphodiesterase 3B and promoting mitochondrial oxidative metabolism, which was largely mimicked by LPE(P-18:0) supplementation, supporting a direct bioactive role for P-LPE (54). In this context, the present study identified a complementary pathway in which PLA2G2F drives the production of P-LPE in the epidermis, connecting plasmalogen metabolism with epithelial–immune signaling and itch responses. Taken together, these studies support a model in which both the generation and degradation of plasmalogen-derived lipids cooperatively regulate tissue-specific signaling pathways, establishing lysoplasmalogens, including P-LPE, as functional lipid mediators rather than passive metabolic intermediates.

Although the precise molecular targets of P-LPE are yet to be identified, several potential mechanisms can be considered. First, P-LPE may act through lipid-sensing G protein-coupled receptors (GPCRs) or nuclear receptors, thereby amplifying the epithelial and immune signaling pathways. Secondly, P-LPE may influence antigen presentation pathways, potentially functioning as a lipid neoantigen presented by CD1d to lipid-reactive immune cells (56). Thirdly, the unique physicochemical properties of plasmalogen-derived lysophospholipids may modulate membrane dynamics. Fourth, P-LPE may influence intracellular signaling pathways such as STAT3 activation (33) and protein kinase A signaling (54). Further studies employing GPCR screening, lipid-protein binding assays, and other target identification approaches will be important to identify the molecular target(s) of P-LPE and clarify its downstream signaling mechanism. Elucidating these mechanisms will expand our understanding of how lipid metabolism interfaces with the immune and sensory pathways in the skin.

Although the present study demonstrates a critical role for the PLA2G2F/P-LPE axis in the MC903-induced model of atopic dermatitis, the MC903 model is characterized by a strong TSLP-dependent type 2 inflammatory response and therefore may not fully recapitulate all aspects of human atopic dermatitis. Future studies using additional experimental models, such as ovalbumin- and house dust mite-induced dermatitis, will be important to generalize the roles of the PLA2G2F/P-LPE axis across distinct pathogenic settings. Such studies will also help clarify the therapeutic potential of targeting this pathway in a broader range of disease contexts. It remains to be elucidated whether long-term inhibition of PLA2G2F would affect physiological skin functions, including cutaneous wound healing and resistance to skin infection, although these important safety considerations are beyond the scope of the present study.

In conclusion, our study revealed the central role of PLA2G2F in coordinating lipid metabolism, immune responses, and itching. This PLA2G2F/P-LPE axis represents a novel epithelial–immune signaling pathway and a potential therapeutic target and biomarker for atopic dermatitis or possibly other skin diseases harboring epidermal–immune crosstalk.

## Data Availability

The original contributions presented in the study are included in the article/**Supplementary Material**, further inquiries can be directed to the corresponding author/s.
