# Supplementary material for: PLA2G2F/lysoplasmalogen axis links epidermal lipid metabolism to type 2 inflammation and itch in atopic dermatitis
Source: Front Immunol. 2026 Sep 7;17:1880255. doi: 10.3389/fimmu.2026.1880255 (PMC13590317; doi:10.3389/fimmu.2026.1880255)
Supplement: Supplementary file 1 [file DataSheet1.pdf]

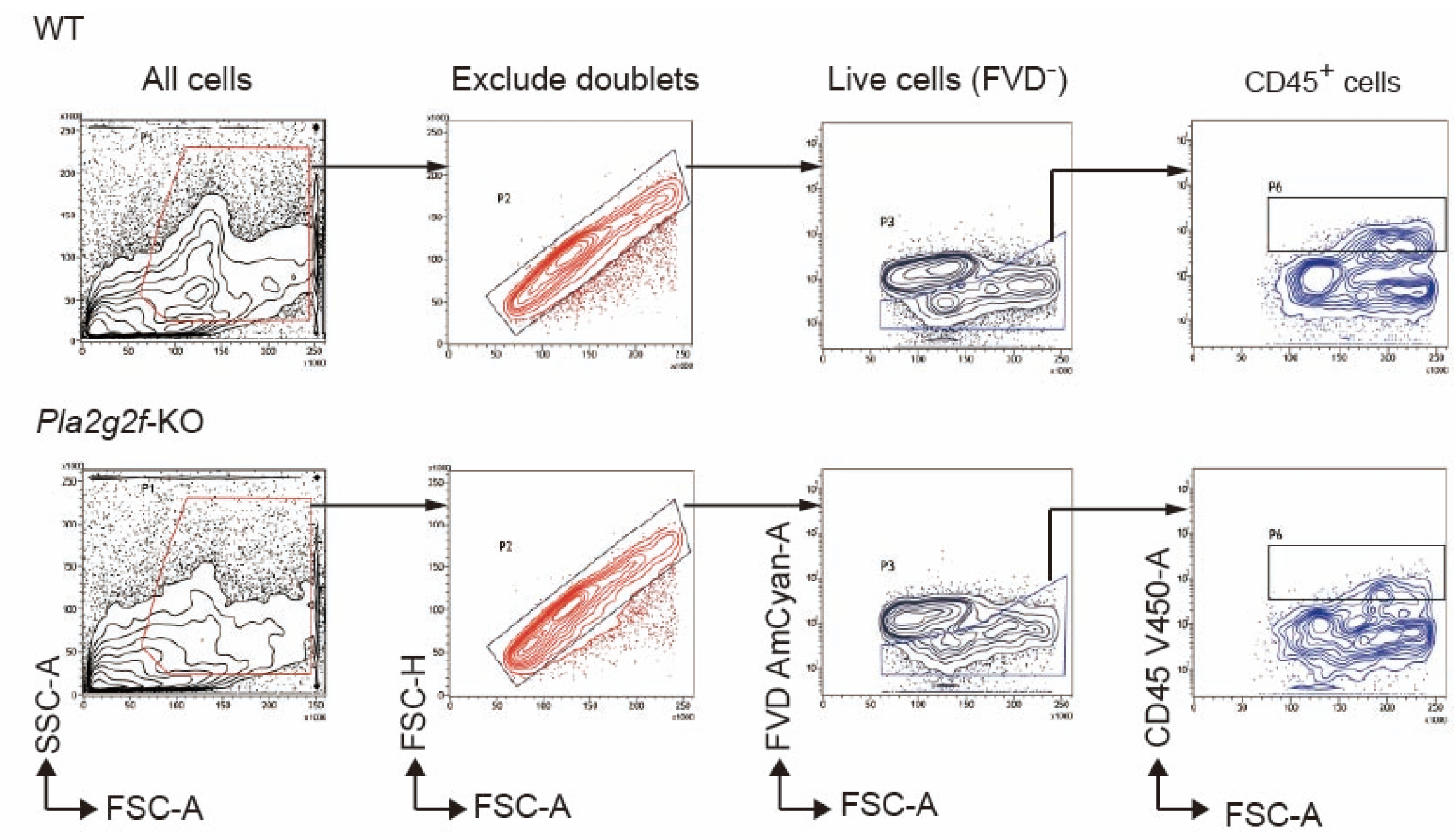

Supplementary Figure S1

Representative flow cytometric gating strategy for CD45⁺ immune cells in the skin of WT and *Pla2g2f*-KO mice (related to Figure 3I). Cells were sequentially gated as follows: all cells (FSC-A vs. SSC-A), singlets (FSC-A vs. FSC-H), live cells (FVD⁻), and finally CD45⁺ immune cells.

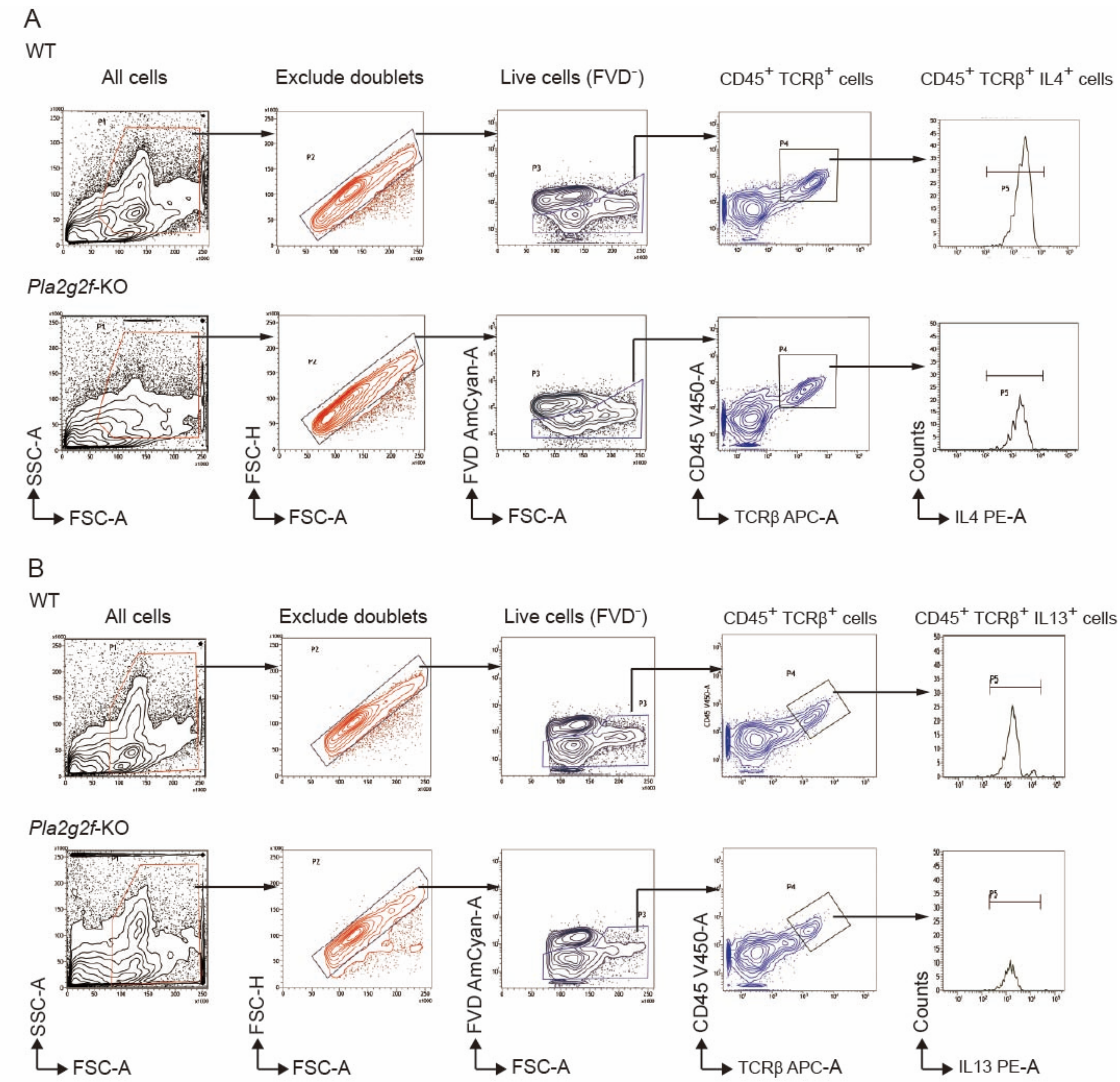

Supplementary Figure S2

Representative gating strategy for IL-4⁺ and IL-13⁺ TCRβ^+ T cells in the skin of WT and *Pla2g2f*-KO mice (related to Figure 3J). CD45⁺ immune cells were further gated on TCRβ^+ T cells, followed by analysis of intracellular IL-4 and IL-13 expression.

Supplementary Table S1. A list of primers used in qPCR analysis by the SYBR Green method.

qPCR was performed using the SYBR Green method with the indicated primers.

Genes	Forward Primers	Reverse Primers
PLA2G2F	5'-CCTGTCCTTCGTGGGCTAC-3'	5'-TTCCTGGTAGCAGCAGTCG-3'
PLA2G1B	5'-TTGTGCTAGCTGTGCTGCTC-3'	5'-TTGATCATTTTGCGGAACTG-3'
PLA2G2A	5'-ACCTGCCCTGTCTCCAAAC-3'	5'-TTTGTTCTGCACTCCTGCTC-3'
PLA2G2D	5'-GCAGCATCTACAAGGACTATTACAGA-3'	5'-CACACCAGCTTCCCTTGTC-3'
PLA2G2E	5'-CACGTGCTGGTGTTCCTTT-3'	5'- TCATCACCCCAAACTGAACC -3'
PLA2G5	5'-GCTCATGGGAGCAGACCTC-3'	5'-CCTTTCATCTCTGGGGTTCTC-3'
PLA2G10	5'-GGCCTCCAGGATATTACGTGTGC-3'	5'-TCAGTCACACTTGGGCGAGTCCG-3'
PLA2G3	5'-ACAGGGTGGCCTAAAACCTC-3'	5'-CAATCTGGTGCTCACACTGG-3'
PLA2G12A	5'-GCGTTCATAAGATAGACACGTACC-3'	5'-CGTGGGAAAGGCTTAGATCC-3'
FLG	5'-GGACTCTGAGAGGCGATCTG-3'	5'-TGCTCCCGAGAAGATCCAT-3'
LOR	5'-TCATCACCCCAAACTGAACC-3'	5'-TCATCACCCCAAACTGAACC-3'
CLDN1	5'-CCTCCTGGGAGTGATAGCAAT-3'	5'-GGCAACTAAAATAGCCAGACCT-3'
IL33	5'-CCACCAAAAGGCCTTCACT-3'	5'-AAGGCAAAGCACTCCACAGT-3'
TSLP	5'-CCAGGCTATTCGGAAACTCA-3'	5'-TCCAGACATTTATTGGTTGTGACT-3'
CCL26	5'-AGTCACAATTGTTTCGGAGTT-3'	5'-AGTCTCCACCTTGGAACTG-3'
IL4R	5'-CTGCTCATGGATGACGTGGTCA-3'	5'-GGTGTGAACTGTCAGGTTTCCTG-3'
IL13RA1	5'-CCTGAATGAGAGGATTTGTCTGC-3'	5'-CAGTCACAGCAGACTCAGGATC-3'
CRLF2	5'-ACGGGGATCTCCTCTATG-3'	5'-GAGGCGTTGGTGTCTCT-3'
IL7R	5'-AGGATGAAAACAAATGGACGCA-3'	5'-CCTTTAAAATAGTGATCAGGGATGG-3'
GAPDH	5'-AGCCACATCGCTCAGACAC-3'	5'-GCCCAATACGACCAAATCC-3'
Hrh1	5'-CTGGTGCTGTATGCAGTGC-3'	5'-GCTACCGACAGGCTGACAA-3'
Hrh4	5'-CCTCGTGGGTTTGATTTCCAT-3'	5'-AGTAATGAGCCAAAACATGCAGA-3'
Trpv1	5'-CCCGGAAGACAGATAGCCTGA-3'	5'-TTCAATGGCAATGTGTAATGCTG-3'
Trpa1	5'-AACGGCTACAGCAGGGAGA-3'	5'-TCCAGGCACATCTTAATCATGTC-3'
Crlf2	5'-TCACGGGGTGATGTCACAGT-3'	5'-AGTGCCATAACGGAACTCCAG-3'
Nefm	5'-CTCGGCTATGCTCAGCTCG-3'	5'-CGGGACAGTTTGTAGTCGCC-3'
Uchl1	5'-AGGGACAGGAAGTTAGCCCTA-3'	5'-AGCTTCTCCGTTTCAGACAGA-3'
Il4ra	5'-AGAAACGCAGGAGATGGACTT-3'	5'-ATGATGATGGCCACCAAGGG-3'
Il13ra	5'-GAAGTGTTCCTGGCTCCCTG-3'	5'-CTCCAGGCTGCTGTACCAATA-3'
Il31ra	5'-TTCAAGACATTGTCAATCAGTGTG-3'	5'-GTCACTGTTTTGATGCTAAGTAGAAGA-3'
Gapdh	5'-GGGTTCCTATAAATACGGACTGC-3'	5'-CCATTTTGTCTACGGGACGA-3'

Supplementary Table S2. A list of primers used in qPCR analysis by the TaqMan method.

qPCR was performed using the TaqMan method with either Roche probe–based assays or TaqMan probe–based assays.

Genes			
	Forward Primers	Reverse Primers	Probe numbers (Roche)
Tslp	5'-CAGCTTGTCTCCTGAAAATCG-3'	5'-AAATGTTTTGTCGGGGAGTG-3'	71
Krt1	5'-TTTGCCTCCTTCATCGACA-3'	5'-GTTTTGGGTCCGGGTTGT-3'	62
Krt14	5'-ATCGAGGACCTGAAGAGCAA-3'	5'-TCGATCTGCAGGAGGACATT-3'	31
Il33	5'-GACACATTGAGCATCCAAGG-3'	5'-AACAGATTGGTCATTGTATGTACTCAG-3'	51
Il4	5'-GAGAGATCATCGGCATTTTGA-3'	5'-TCTGTGGTGTTCTTCGTTGC-3'	2
Il31	5'-CCATGATCTTCCACACAGGAA-3'	5'-GCACCGAAGGACAAGCTG-3'	20
	Taqman probes (Applied Biosystems) accession No.		
Pla2g2f	Mm00478872_m1		
Gapdh	Taqman Rodent GAPDH control reagents (4308313)		
